# Poor awareness of syphilis prevention and treatment knowledge among six different populations in south China

**DOI:** 10.1186/s12889-016-2966-4

**Published:** 2016-03-28

**Authors:** Xiaobing Wu, Fuchang Hong, Lina Lan, Chunlai Zhang, Tiejian Feng, Yingzhou Yang

**Affiliations:** Department of STD control and prevention, Shenzhen Center for Chronic Disease Control, No. 2021, Buxin Road, Luohu District, Shenzhen City, Guangdong Province P.R. China

**Keywords:** Syphilis, Awareness, Knowledge, Homosexuality, Education

## Abstract

**Background:**

China is facing an emerging epidemic of syphilis, and the cities in south China are most affected. Knowledge is a key factor in the prevention of syphilis infection, however, little is reported about how much people know about syphilis. This study was aimed at assessing peoples’ awareness status in a city located in south China.

**Methods:**

Six populations were recruited for this study, including urban residents, factory workers, college students, pregnant women, female sex workers (FSWs), and men who have sex with men (MSM). A questionnaire designed by the National Center for Disease Control and Prevention was used to assess participants’ awareness of syphilis knowledge. About 5 % of participants were randomly selected to conduct a telephone survey for confirming the validity of fieldwork.

**Results:**

The study recruited 3470 participants, and 61.2 % of participants were assigned to the awareness group. College students had the smallest percentage of awareness at 51.7 % (371/718), followed by FSWs at 53.9 % (200/371), factory workers at 56.0 % (381/679), urban residents at 65.4 % (435/665), pregnant women at 66.0 % (451/683), and MSM at 81.1 % (287/354). Multivariate logistic regression analysis showed that MSM and FSWs—but not factory workers and pregnant women—had more awareness of syphilis knowledge when comparing with urban residents; however, college students presented less awareness of syphilis knowledge than urban residents. Participants of younger age, of female gender, with lower education levels and without Shenzhen *hukou* possessed less awareness of syphilis knowledge than those of older age, of male gender, with higher education levels and with Shenzhen *hukou* respectively.

**Conclusions:**

The percentages of awareness on syphilis knowledge found in this study are far from the benchmark set in the national 10-year plan. Tailored interventions for different subgroups to increase syphilis awareness are urgently warranted.

**Electronic supplementary material:**

The online version of this article (doi:10.1186/s12889-016-2966-4) contains supplementary material, which is available to authorized users.

## Background

Syphilis is a sexually transmitted infection caused by *Treponema pallidum*. People with untreated infection might present the symptoms not only limited to ulcer, chancre, skin rash, mucocutaneous lesions and lymphadenopathy, but also cranial nerve dysfunction, auditory or ophthalmic abnormalities, and cardiac or gummatous lesions [1]. Active syphilis infection in pregnancy when untreated or inadequately treated, can lead to abortion, stillbirth, prematurity, low birthweight, neonatal death or congenital syphilis [2]. Syphilis can also increase the risk of HIV acquisition and transmission [3]. Regular treatment for syphilis patients is thus suggested to be one way in controlling HIV epidemic.

Syphilis remains a public health problem worldwide. According to the World Health Organization (WHO) estimates, more than 12 million people infected with syphilis every year, most of whom lived in the developing countries [4]. China has been experiencing a syphilis epidemic over the past two decades, showing a rise of incidence from 6.4 per 100,000 in 2000 to 32.9 per 100,000 in 2013 [5, 6]. The cities in south China have witnessed this alarming increase [7, 8]. Shenzhen, a special economic zone located in south China and with a population of over 10 million, is one of the cities most affected by syphilis. The city reported its first syphilis case in the early 1980s, while the number was around 7000 in 2013 with an incidence of 64.0 per 100,000, which was about 2 times higher than the national incidence in the same year [9, 10]. The high syphilis incidence in south China provides challenges to current syphilis prevention and intervention strategies and measures.

An important task for decision makers in disease control is to understand the factors that influence people’s health behavior for the management and prevention of the disease. Much research on health behaviors has focused on the perceptual and cognitive factors that underlie people’s motivation or intention to attend health care appointments or adopt behaviours that are proposed to avoid the infection [11–13]. People’s view towards a disease will guide coping behaviours and finally affect the health outcomes. Given the epidemic of syphilis in China and even worldwide, however, little is reported about how much people know about syphilis [14]. This study aims to fill in this information gap.

## Methods

### Participants

The study targeted six different populations: urban residents, factory workers, college students, pregnant women, female sex workers (FSWs), and men who have sex with men(MSM). An urban resident was defined as a person aged 18–49 years who had been living in the city for ≥1 year. Factory workers were defined as those aged 18–49 years, hired by factories in Shenzhen and had been living in the city for < 1 year. College students were those currently studying in college or junior college. Pregnant women were defined as those aged ≥18 years who had visited antenatal clinics for health care services. FSWs were defined as women who aged ≥18 years, worked in entertainment venues (including karaoke bar, hair salon, sauna, bathroom and hotel), and were likely to have commercial sex behaviors according to the gatekeepers. MSM were defined as men aged ≥18 years who had reportedly engaged in anal sex during the last year.

### Study design

This study was based on primary survey data that our research team collected in Shenzhen from July 2012 to December 2012. The study was approved by the Institutional Review Board and Human Research Ethics Committee of Shenzhen Center for Chronic Disease Control (SZCCC). The local government also agreed with this study. The six targeted populations were interviewed as follows. For the interview of urban residents, 3 of 10 districts in Shenzhen were randomly selected. Two neighborhoods from each district were selected and two communities from each selected neighborhood were involved. Approximately 55 families from each community were selected, and one member per family was included in the study. For the interview of factory workers, two districts where factories concentrated were selected. Three factories from each selected district were involved and about 110 workers from each factory were recruited. Students were recruited from one college and one junior college located in south of Shenzhen. About 350 students from each college were interviewed. For pregnant women, 3 of 10 districts in Shenzhen were selected. One antenatal care clinics from each selected district was involved and about 220 pregnant women from each clinic were recruited. The survey for FSWs and MSM was integrated into the HIV surveillance and prevention program. FSWs were recruited from the entertainment venues, while MSM were recruited from gay venues. Urban residents, pregnant women, FSWs and MSM were interviewed face to face, while students and factory workers completed the questionnaire by themselves. Before the interviews, participants were asked if they had ever involved in the similar study, and those with ‘yes’ answer would be excluded. Participants were explained that this study was anonymous and confidential, the data would be used strictly for academic purposes, and they had their right to withdraw from the interview anytime during the interview. A brief introduction of study purpose and participants’ right was showed at the beginning of the questionnaire. Verbal informed consent was obtained from the participants for the publication of this report and any accompanying images. 

All the investigators were trained at the SZCCC. In order to confirm the validity of the fieldwork, after the fieldwork was completed, 5 % of the participants in each target group were randomly selected for call backs in order to compare the consistency of their answers between fieldwork and telephone interviews. If the participant could not be reached via telephone after three attempts on different days, or if the participants refused to answer the call, an alternative participant was selected instead.

### Measures

The questionnaire used in this study was designed by the National Center for Disease Control and Prevention (CDC) and was divided into three parts. The first part was about participants’ socio-demographic characteristics, including age, gender, ethnicity, education level, *hukou* status (a common name used in mainland China for the household registration system, which is linked to basic welfare services, social benefits, and other subsidized government-provided services), and telephone number (if they agreed to join the telephone interview). The second part contained eight items for measuring syphilis prevention and treatment knowledge, including respects of transmission routes (items 1, 7, and 8), curative effect (item 2), asymptomatic appearance (item 3), prevention measures (item 4), relationship between HIV and syphilis (item 5), and partner notification and screening (item 6). According to the calculation method described by the National CDC and published papers [15], participants who answered six or more items correctly were assigned to the ‘awareness’ group. The awareness status was considered to be the dependent variable in this study (0 = no, 1 = yes). The percentage of awareness was calculated using cases of awareness divided by the total targeted cases. The third part contained one item assessing the participants’ source of acquiring knowledge on syphilis. The answer to this question included promotion activities, television, broadcast, newspapers and journals, books, friends and companions, health staffs, brochures and leaflets, internet, and lectures. The questionnaire for telephone survey only contained eight items to measure syphilis knowledge and 3 items of socio-demographic variables to confirm participants’ identity. The dataset supporting the results of this article is included within the article Additional file 1.

### Data analysis

Epidata 3.0 software (Epidata association, Denmark) was used to entry data by two students, and SPSS 16.0 (SPSS Inc., Chicago, IL, USA) was used to analyze the data. Descriptive statistics were used to describe participants’ demographic characteristics and percentages of participants assigned to awareness group. Logistic regression analysis was used to identify factors that were associated with the awareness status. Odds ratio (OR) and its 95 % confidential interval (95 % CI) were calculated. Kappa test was used to compare the consistency of the awareness status between fieldwork and the telephone survey among the selected participants.

## Results

### Demographic characteristics

This study recruited a total of 3470 participants, including 665 urban residents, 679 factory workers, 718 college students, 683 pregnant women, 371 FSWs and 354 MSM. The average age of the participants was 28 years (standard deviation [SD], 8.2 years). Among all participants, 37.1 % were men; 95.3 % were Han; 42.9 % had received college or higher education; and 24.2 % were with Shenzhen *hukou*.

### Awareness of syphilis prevention and treatment knowledge

Table 1 presents the responses to the questions on syphilis among different populations. The highest proportion of participants (91.0 %) correctly responded that sex partners of syphilis patients should attend a hospital for serological examination. More than three quarters of participants understood that syphilis is mainly transmitted through sexual contact (78.6 %) and using condoms in sexual contact can prevent syphilis transmission (76.0 %). Less than 60 % of the participants correctly answered that syphilis infection can increase the risk of HIV transmission and infection (54.7 %).

**Table 1:** Awareness of individual items of syphilis knowledge

Item	Total		Urban residents		Factory workers		College students		Pregnant women		FSWs		MSM	
	Frequency	%	Frequency	%	Frequency	%	Frequency	%	Frequency	%	Frequency	%	Frequency	%
1	2727	78.6	564	84.8	516	76.0	529	73.7	531	77.7	273	73.6	314	88.7
2	2263	65.2	477	71.7	431	63.5	400	55.7	425	62.2	219	59.0	311	87.9
3	2074	59.8	390	58.6	352	51.8	472	65.7	430	63.0	184	49.6	246	69.5
4	2636	76.0	544	81.8	471	69.4	509	70.9	559	81.8	251	67.7	302	85.3
5	1897	54.7	348	52.3	414	61.0	314	43.7	379	55.5	195	52.6	247	69.8
6	3157	91.0	609	91.6	624	91.9	618	86.1	644	94.3	326	87.9	336	94.9
7	2382	68.6	492	74.0	453	66.7	370	51.5	558	81.7	231	62.3	278	78.5
8	2464	71.0	498	74.9	417	61.4	519	72.3	520	76.1	244	65.8	266	75.1

Item 1: Syphilis is mainly transmitted through sexual contact (True)

Item 2: Syphilis is curable (True)

Item 3: A man looks healthy may have syphilis (True)

Item 4: Using condoms correctly in sexual contact can prevent syphilis transmission (True)

Item 5: Syphilis infection can increase the risk of HIV transmission or acquisition (True)

Item 6: Sex partners of syphilis patients need to attend a hospital for serological examination (True)

Item 7: Syphilis infected women can transmit the syphilis to their neonatal (True)

Item 8: Having dinner or shaking hands with syphilis patients can infect syphilis (False)

Overall, 61.2 % of participants responded six or more items correctly and considered to be ‘awareness’. The percentage for urban residents, factory workers, college students, pregnant women, FSWs, and MSM were 65.4, 56.0, 51.7, 66.0, 53.9, and 81.1 %, respectively. The results from univariate logistic regression model showed that the population, age, gender, education level, and *hukou*—but not ethnicity—were associated with awareness status at a significance level of 0.05 (Table 2). In the multivariate logistic regression model, FSWs and MSM—but not factory workers and pregnant women—had more awareness of syphilis knowledge when comparing with urban residents; however, college students presented less awareness of syphilis knowledge than urban residents. Participants of younger age, of female gender, and with lower education levels possessed less awareness of syphilis knowledge than those of older age, of male gender, and with higher education levels respectively. Participants without Shenzhen *hukou* (from other cities in Guangdong province and from other provinces in mainland China) had less awareness of syphilis knowledge than those with Shenzhen *hukou* (Table 2).

**Table 2:** Factors associated with the awareness status of syphilis knowledge in logistic regression analysis

	Number of participants	Awareness of syphilis knowledge		OR_u_	(95 % CI)	OR_m_	(95 % CI) ^a^
		Frequency	%				
Population							
Urban residents	665	435	65.4	1.00		1.00	
Factory workers	679	380	56.0	0.67	(0.54–0.84)^***^	1.08	(0.84–1.39)
College students	718	371	51.7	0.57	(0.46–0.70)^***^	0.45	(0.32–0.64)^***^
Pregnant women	683	451	66.0	1.03	(0.82–1.29)	0.99	(0.76–1.29)
FSWs	371	200	53.9	0.62	(0.48–0.80)^***^	1.38	(1.02–1.88)^*^
MSM	354	287	81.1	2.26	(1.66–3.09)^***^	1.59	(1.13–2.24)^**^
Age (years)							
≤ 20	686	346	50.4	1.00		1.00	
21–25	884	468	52.9	1.11	(0.91–1.35)	1.03	(0.82–1.30)
26–30	846	582	68.8	2.17	(1.76–2.67)^***^	1.76	(1.33–2.33)^***^
31–35	452	314	69.5	2.24	(1.74–2.87)^***^	1.79	(1.31–2.45)^***^
> 35	602	414	68.8	2.16	(1.72–2.72)^***^	1.64	(1.20–2.25)^**^
Gender							
Male	1289	880	68.3	1.00		1.00	
Female	2181	1244	57.0	0.62	(0.53–0.71)^***^	0.64	(0.54–0.77)^***^
Ethnicity							
Han	3307	2022	61.1	1.00			
Others	163	102	62.6	1.06	(0.77-1.47)		
Education							
Junior middle school or lower	1042	542	52.0	1.00		1.00	
Senior middle school	939	613	65.3	1.73	(1.45–2.08)^***^	1.67	(1.38-2.02)^***^
College or above	1489	969	65.1	1.72	(1.46–2.02)^***^	2.66	(2.07-3.42)^***^
Hukou							
Shenzhen	839	572	68.2	1.00			
Other cities in Guangdong province	936	543	58.0	0.64	(0.53–0.78)^***^	0.79	(0.64–0.97)^*^
Other provinces in China	1645	970	59.0	0.67	(0.56–0.80)^***^	0.67	(0.54–0.84)^***^
Hong Kong, Macao, and Taiwan	50	39	78.0	1.65	(0.83–3.28)	1.10	(0.53–2.29)

*CI* Confidential interval, *OR*_*u*_ odds ratio in univariate analysis, *OR*_*m*_ odds ratio in multivariate logistic regression

*p < 0.05; **p < 0.01; ***p < 0.001

^a^All the variables with *p* < 0.05 in univariate analysis were selected stepwise via a forward method, to be entered into the multivariate logistic regression model

### Source of knowledge

Among the 10 sources surveyed, television was reported as the most common means for acquiring syphilis prevention and treatment knowledge (58.5 %), followed by promotional activities (51.4 %), internet (41.2 %), brochures/leaflets (38.4 %), books (36.7 %), and newspapers/journals (36.3 %). A small percentage of FSWs and MSM reported receiving the knowledge from doctors (28.3 and 33.6 %, respectively) and friends/companions (27.2 and 28.2 %, respectively).

### Consistency of awareness status between fieldwork and telephone survey

Among all participants, 70.0 % agreed to join the telephone survey. In total, 180 subjects were randomly selected to join the telephone survey (5.2 % of all participants). The percentages of awareness for the fieldwork and telephone survey were 62.2 and 70.6 %, respectively. The Kappa value for this was 0.44 (*p* < 0.001), indicating the consistency was acceptable and the survey data were reliable.

## Discussion

Given the large burden of syphilis and the potentially severe consequences of syphilis infection, the Chinese Ministry of Health recently announces a 10-year plan for syphilis prevention and control. This 10-year plan advocates six special benchmarks for evaluating the success of the national syphilis control programs, and the awareness of syphilis prevention and treatment knowledge is one of the six important goals [16]. This study demonstrates the poor awareness of syphilis knowledge among six different populations. The percentages of awareness in this study range from 51.7 to 81.1 %, which are far from the 2020 goals in the 10-year plan of 85–90 % for the general populations and 95 % for high-risk populations. Similar results were found in other cities located in south China, such as Guangzhou (provincial capital of Guangdong, percentage of awareness among different populations ranged from 53.7 to 90.4 %) [15]. The large gap between the poor awareness observed in the study and the goal in the 10-year plan may explain why the south China experiences the syphilis epidemic, and the study results raise big challenges to the current syphilis control programs in south China.

Our result that college students possess the least awareness of syphilis knowledge is similar to the finding of study conducted in Guangzhou (56.5 %) [15]. Several empirical studies reported that college students in China were sexually active and a few of them were involved in commercial sex, but only half had ever received HIV/STI interventions or services [17, 18]. In this study, a part of students have never heard about syphilis, about one-fourth do not know they can get the infection via sex behaviors, and about 30 % do not know using condom correctly can prevent the infection. The lack of knowledge may stem partly from limited sexual education programs and classes in schools as well as social norms that discourage discussion about sex and reproductive health [19]. Knowledge is a key factor in the prevention of HIV/STI infection since it associated with self-protection behaviors as well as misconceptions about vulnerability to infection [20]. The poor awareness of syphilis knowledge found among college students indicating the necessity of building up a teaching platform or health service to raise their awareness.

MSM and FSWs are considered to be high risk groups in syphilis infection, and they use to be showed with high awareness of HIV knowledge [21, 22]. To our surprise, only MSM, but not FSWs, display high percentage of awareness on syphilis prevention and treatment knowledge. This may be explained in three ways. Firstly, more and more intervention programs focus on MSM to promote their safe sex behaviors as well as their knowledge towards HIV/STI, but limited programs were conducted among FSWs [22, 23]. Secondly, the majority of MSM have high education levels (approximately 80 % had received senior middle school or higher education in this study), and they can search disease information by themselves. On the contrary, most of FSWs have low education levels (nearly 70 % only finished junior middle school or below). After adjusting for the education levels, this study finds FSWs have a favorable awareness of syphilis when comparing with urban residents (the value of OR is larger than 1). Thirdly, many MSM are from Hong Kong where more education and intervention programs for this population were conducted [24, 25]. FSWs are mainly from other provinces in mainland China, where interventions are insufficient.

The finding that participants who do not have Shenzhen *hukou* are likely to have less syphilis knowledge may be due to their limited accessibility to preventative interventions as well as their socioeconomic vulnerabilities. Researchers have already pointed out that China’s current household register system plays an important role in the allocation of economic resources, educational opportunities, and other welfare benefits for migrants [26]. Many labor migrants reside in sub-standard and crowded housing conditions, experience economic hardship and are poorly assimilated into their host communities [20]. Most of migrants concentrate in neighborhoods and workplace that consist mostly of other migrants, with few opportunities to interact with non-migrants and seldom take part in social activities [27]. Several studies from China have shown that subsets of rural-to-urban migrants are lack of HIV/STI knowledge, have increased likelihood of risky sexual behaviors [28, 29], and are more likely to have primary or secondary syphilis [9]. Moreover, their low income and poor awareness create barriers for their ability to seek timely medical service, which implies more transmission if they get the disease.

To our knowledge, the 8 items adopted in this study represent the first questionnaire used nationwide to assess peoples’ awareness of syphilis knowledge. Examinations of individual item scores show that a certain proportion of participants have misconceptions about syphilis. About 45 % of participants do not know syphilis can increase the risk of HIV transmission and acquisition. Previous studies have pointed out that syphilitic ulcers would disrupt epithelium and mucosa, thus aiding the passage of HIV. Syphilis infections may also promote HIV replication, which increase the risk of transmission from HIV-positive patients to HIV-negative individuals [3, 30]. Epidemiological studies have observed the risk of HIV acquisition among persons with syphilis is 2.5 to 10 times as high as that among those without syphilis [31, 32]. Timely and standardized treatment for syphilis patient is thus considered to be an effective way to control HIV epidemic.

Finally, our finding shows that people acquire syphilis knowledge from different sources. Television and promotional activities are still two main routes, but more and more participants prefer to search for information on the internet or electronic media. Some MSM and FSWs acquire relevant knowledge from their doctors, friends or companions. Given that the awareness of syphilis knowledge and information resources varied among different populations, developing tailored syphilis messaging interventions targeting both high risk and low risk population is an important goal. In last decade, China has set up a good platform to deliver HIV prevention knowledge among different populations. The percentages of awareness for HIV knowledge among migrants and MSM are above 90 %, which are much higher than that of syphilis knowledge. Therefore, the 10-year plan advocates for local governments to make use of this platform and integrate syphilis prevention knowledge into the platform. This integration strategy allows us to combat syphilis and HIV epidemic with current limited financial support and human resources.

### Limitations

This study is limited by its reliance on a convenience sample on FSWs and MSM. As is known to all, FSWs and MSM are hard to reach and it is nearly impossible to randomly sample this group. But for other four groups of participants, this study used stratified sampling method to ensure the representativeness. Second, no data was collected on risk behaviors, which is a well-established determinant of syphilis infection. Further study should be conducted to confirm the associations between the awareness of syphilis knowledge and risk behaviors.

## Conclusions

This study demonstrates poor awareness of syphilis prevention and treatment knowledge among six populations in Shenzhen, China. Health education programs and health promotion strategies targeting different subgroups are warranted to increase the awareness in south China in order to combat the currently emerging epidemic.

## Additional file

Additional file 1: Dataset of the syphilis awareness survey among six different populations in Shenzhen, China. (XLS 934 kb)
